# A review of sepsis-induced cardiomyopathy

**DOI:** 10.1186/s40560-015-0112-5

**Published:** 2015-11-11

**Authors:** Ryota Sato, Michitaka Nasu

**Affiliations:** Department of Emergency and Critical Care Medicine, Urasoe General Hospital, 4-16-1, Iso, Urasoe, Okinawa Japan

**Keywords:** Sepsis-induced cardiomyopathy, Septic cardiomyopathy, Cardiac depression in sepsis

## Abstract

Sepsis-induced cardiomyopathy is a reversible myocardial dysfunction that typically resolves in 7–10 days. It is characterized by left ventricular dilatation and depressed ejection fraction. However, many uncertainties exist regarding the mechanisms, characteristics, and treatments of this condition. Therefore, this review attempts to summarize our current knowledge of sepsis-induced cardiomyopathy.

## Introduction

**Fig. 1:**
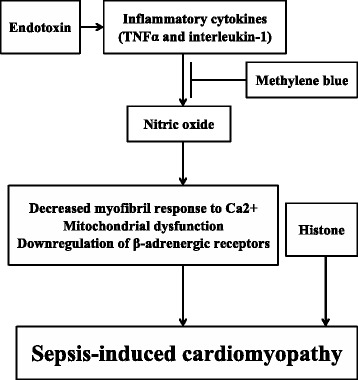
Mechanisms of sepsis-induced cardiomyopathy. Endotoxins cause depressed cardiac contractility, which is mediated by enhanced nitric oxide (NO) production. Tumor necrosis factor and interleukin-1β also contribute to NO overproduction. NO is believed to act in the heart by decreasing myofibril response to calcium, inducing mitochondrial dysfunction, and downregulating β-adrenergic receptors. These reactions lead to sepsis-induced cardiomyopathy. Methylene blue, an inhibitor of the NO pathway, counteracts the myocardial depression. Histones occur inside the nucleus and can be released into circulation because of extensive inflammation and cellular death during sepsis. Since cardiac dysfunction can be ameliorated by anti-histone antibodies in a septic mouse model, histones may be implicated in the pathophysiology of sepsis-induced cardiomyopathy

Sepsis is a dysregulated systemic inflammation caused by infections involving various organs. Sepsis-induced cardiomyopathy is a complication of severe sepsis and septic shock first described by Parker et al. in 1984 as a reversible myocardial depression that occurs in patients with septic shock [1]. In sepsis-induced cardiomyopathy, the myocardium is functionally and structurally injured by inflammatory cytokines and mitochondrial dysfunction. However, our understanding regarding this condition remains incomplete. Recently, the development of tools, including echocardiography, has made it possible to visualize the hemodynamics of sepsis-induced cardiomyopathy. Sepsis-induced cardiomyopathy has three characteristics: left ventricular dilatation, depressed ejection fraction, and recovery in 7–10 days. Also, advances in molecular biology have made understanding the mechanisms of sepsis-induced cardiomyopathy possible (Fig. 1). Chemical mediators, including endotoxins, cytokines, and nitric oxide, appear to be the main mediators of sepsis-induced cardiomyopathy. The treatment strategy of sepsis-induced cardiomyopathy is the same with the adequate treatment of sepsis without cardiomyopathy. Although dobutamine is recommended in current guidance, recent trials have demonstrated that in patients with sepsis, it does not improve the prognosis and may have adverse effects. Here, we discuss the mechanisms, characteristics, and treatments of sepsis-induced cardiomyopathy.

## Review

### Characteristics of sepsis-induced cardiomyopathy

In 1984, Parker et al. reported decreased ejection fraction and increased end-diastolic volume in septic shock survivors. These changes in left ventricular function were of rapid onset and reversed over 7–10 days in survivors; however, they were less profound in those who died [1, 2]. Moreover, Vieillard et al. demonstrated the mortality rate among patients in a hyperkinetic state to be significantly higher than that among patients in either a hypokinetic or normal-output state (100 vs 43 and 24 %) [3]. Conversely, a recent meta-analysis suggested that ventricular dysfunction or dilatation in patients with sepsis was not associated with lower mortality [4]. It suggested that mortality does not depend on whether the patients have sepsis-induced cardiomyopathy or not; however, it depends on whether the patient’s heart is hyperkinetic or not. Because patients with sepsis-induced cardiomyopathy tend to be either hypokinetic or normokinetic but not hyperkinetic, in patients with sepsis-induced cardiomyopathy, the outcomes may be better than in patients without sepsis-induced cardiomyopathy. Larger studies are needed to clarify whether sepsis-induced cardiomyopathy is associated with improved outcomes.

Sepsis also triggers takotsubo cardiomyopathy, also known as stress cardiomyopathy, apical ballooning syndrome, or broken-heart syndrome, which is different from sepsis-induced cardiomyopathy. Takotsubo cardiomyopathy typically occurs when the contractile function of the mid-to-apical segments of the left ventricle is depressed and there is hyperkinesis of basal walls, producing a balloon-like appearance of the distal ventricle. Several studies have reported that this syndrome is induced by increased catecholamine levels. In takotsubo cardiomyopathy, the left ventricular function usually returns to normal within a few weeks. At a cellular level, changes were shown to be reversible with normalization of the left ventricular ejection fraction, including the cytoskeletal protein derangements, increase in extracellular matrix proteins, and accumulation of intracellular glycogen [5]. Although sepsis and septic shock can trigger takotsubo cardiomyopathy, the etiology and myocardial alterations appear to be different from those of sepsis-induced cardiomyopathy. In sepsis-induced cardiomyopathy, there is global ventricular dysfunction and dilated left ventricle without regional dysfunction. In takotsubo cardiomyopathy, there is typically regional dysfunction, especially characterized by apical ballooning mimicking acute coronary syndrome. Therefore, the accurate diagnosis of takotsubo cardiomyopathy requires coronary angiography to rule out acute coronary syndrome. In addition, many reports on takotsubo cardiomyopathy have mentioned various underlying diseases; this suggests that the pathophysiology of takotsubo cardiomyopathy is not specific to one particular disease. For these reasons, sepsis-induced cardiomyopathy and takotsubo cardiomyopathy are two different entities.

Despite the lack of diagnostic criteria for sepsis-induced cardiomyopathy to date, it is known to have three characteristics. The first is left ventricular dilatation with normal or low filling pressure. This probably occurs due to an increase in the left ventricular compliance, which was first described in patients with septic shock in 1984 [1]. Later, another study of the left ventricle response to volume loading showed that there was an abnormal increase in left ventricular end-diastolic volume in sepsis survivors, implying increased ventricular compliance [6]. The second characteristic is depressed ejection fraction. Parker et al. reported that end-diastolic and systolic ventricular volumes were increased but with normal or elevated stroke volume and cardiac index in septic shock survivors. In this study, although the number of patients is less, these results suggest that decreased ejection fraction may be caused by ventricular dilatation and not by decreased stroke volume. Because ejection fraction is defined as the stroke volume divided by the end-diastolic ventricular volume, the denominator increases as the ejection fraction decreases [7]. Vincent et al. demonstrated that the left and right ventricular ejection fractions are depressed in patients with septic shock [8]. Other studies support this result and have shown the development of right ventricular dilatation [6, 9, 10]. The third characteristic of sepsis-induced cardiomyopathy is that it should normalize within 7–10 days [1, 11, 12]. In sepsis-induced cardiomyopathy diagnosis, the first and second characteristics are particularly important and easy to detect using echocardiography. Thus, echocardiography in sepsis management is the most important thing for diagnosing sepsis-induced cardiomyopathy.

B-type natriuretic peptide (BNP), a diuretic hormone, is released from ventricular myocardium in response to cardiac wall stretch. Recent observational study demonstrated that sepsis-induced cardiomyopathy is associated with BNP rise, although not independently, whereas left ventricular filling pressures do not correlate with the BNP levels [13]. In this study, the author concluded that the severity of critical illness, rather than sepsis-induced cardiomyopathy, is probably the main determinant of BNP rise in critical patients with sepsis. For this reason, BNP should not be used as a predictive marker of sepsis-induced cardiomyopathy.

Troponin is a very sensitive and specific protein of myocardial damage and often used for the diagnosis of acute coronary syndrome (ACS). Troponin elevation is common in septic shock patients, and it was estimated that 43–85 % of patients with sepsis showed cardiac troponin I elevation [14–16]. Bessiere et al. performed a meta-analysis and reported that troponin elevation is associated with a higher risk of death among patients with sepsis [17]. However, the use of troponin to diagnose sepsis-induced cardiomyopathy is limited because there are many causes such as ACS and low renal function that affect troponin levels.

### The mechanisms of sepsis-induced cardiomyopathy

Two possible causative mechanisms have been proposed to explain sepsis-induced cardiomyopathy. First, myocardial ischemia resulting from inadequate coronary blood flow has been proposed on the basis of a study in animals [18]. Second, there are strong arguments that chemical mediators, such as endotoxins, cytokines, and nitric oxide, are causative. To determine whether myocardial depression in humans with septic shock was associated with reduced coronary flow, a study was conducted that used coronary sinus thermodilution catheters to measure coronary flow and myocardial metabolism in seven patients [19]. Myocardial depression was observed in four of the seven patients who had coronary flow similar to or higher than that of controls and similar to that of other three patients. Therefore, reduced coronary flow may not contribute to the pathogenesis of sepsis-induced cardiomyopathy.

Concerning the role of chemical mediators, there are stronger arguments to support their role in sepsis-induced cardiomyopathy. Anthony et al. demonstrated that endotoxin administration to controls caused the left ventricular function to become depressed [20], with left ventricular end- and end-systolic volume indexes increasing by 18 and 24 %, respectively. Flesch et al. also demonstrated that exposure of the myocardium to endotoxins caused depressed cardiac contractility, which was mediated by enhanced inducible nitric oxide synthase activity and nitric oxide release [21]. In 1985, Parriio et al. demonstrated in vitro that myocardial cell shortening was reduced by exposure to the serum of patients with sepsis [22]. The same team later showed that this response was caused by tumor necrosis factor alpha (TNF-α) [23], which was confirmed when Vincent et al. demonstrated that anti-TNF antibody administration improved ventricular function without changing the cardiac filling pressure [24]. In a more recent study, interleukin-1β has also been implicated [25], further supporting cytokine’s role in sepsis-induced cardiomyopathy.

Kumar et al. have suggested that cytokine’s effect on cardiac myocytes results from an increase in both intracellular cyclic guanosine monophosphate and nitric oxide [26]. Because the half-lives of TNF and interleukin-1β are less than 6 h, nitric oxide appears to have an important contributory role in the pathogenesis of sepsis-induced cardiomyopathy. Nitric oxide is thought to act in the heart by decreasing myofibril response to calcium [27], inducing mitochondrial dysfunction [28], and downregulating β-adrenergic receptors [28, 29]. Some studies reported that the severity of cardiac dysfunction and mortality can be related to nitric oxide overproduction and mitochondrial dysfunction [30–32]. Larche et al. demonstrated that mitochondrial dysfunction in sepsis is causative rather than epiphenomenal and relevant in terms of myocardial dysfunction in sepsis [33]. In 2001, Kirov et al. evaluated the effects of continuous infusion of methylene blue, an inhibitor of the nitric oxide pathway, on the hemodynamics and organ function of patients with septic shock. They reported that continuous infusion of methylene blue counteracted the myocardial depression, maintained oxygen transport, and reduced the need for concurrent adrenergic support [34]. Later, in a systematic review, Kwok et al. reported that methylene blue administration during sepsis increased the mean arterial pressure and systemic vascular resistance while decreasing the vasopressor requirement [35].

A recent study demonstrated that high-circulating histone levels were significantly associated with new-onset left ventricular dysfunction and arrhythmias in patients with sepsis with no previous cardiac dysfunction [36]. However, because histones occur inside the nucleus and can be released into circulation because of extensive inflammation and cellular death during sepsis, it is unclear whether the circulating histones are the cause or the result of sepsis-induced cardiomyopathy. Further research is warranted to decide the role of circulating histones in the pathogenesis of sepsis-induced cardiomyopathy.

Although the reason why sepsis-induced cardiomyopathy resolves within 7–10 days is poorly understood, the mechanisms proposed in this section appear to be critical. In particular, chemical mediators, such as endotoxins and cytokines, are currently regarded as the most likely cause of sepsis-induced cardiomyopathy. However, the mechanism of myocardial recovery in sepsis-induced cardiomyopathy is poorly understood, and further research is clearly needed.

### Treatments for sepsis-induced cardiomyopathy

In 2001, Rivers et al. reported that early goal-directed therapy was effective for severe sepsis management. Although this strategy has become a standard therapy worldwide, the original study was only a small single-center study [37]. The ProCESS [38] and ARISE [39] trials (in 2014) and the ProMiSe trial [40] (in 2015) have since demonstrated that early goal-directed therapy did not improve outcomes compared with usual care. There is a widespread agreement that standard treatment for sepsis should focus on infection control and optimization of hemodynamic parameters by fluid resuscitation and vasopressor therapy. This strategy is also recognized as the standard therapy for sepsis-induced cardiomyopathy. Noradrenaline is recommended as a first-line vasopressor; however, some studies reported that vasopressin also may be effective for septic shock. Russell et al. suggested that low-dose vasopressin may reduce the mortality of patients with less severe septic shock, although there was no significant difference between vasopressin and noradrenaline in the 28- and 90-day mortality of all patients with septic shock [41]. From this finding, current guidance describes that low-dose vasopressin can be added to noradrenaline with the intent of either raising mean arterial pressure or decreasing noradrenaline dosage; however, low-dose vasopressin is not recommended as a single vasopressor [42]. Mehta et al. reported that troponin, CK, and ECG are not different in patients with septic shock who are treated with noradrenaline and vasopressin [43]. This finding suggests that vasopressin also may be effective for the management of sepsis-induced cardiomyopathy. However, further study is warranted to confirm the effectiveness of vasopressin.

Current guidance recommends using dobutamine [42] to increase the cardiac index [44]. However, Gattinoni et al. reported that hemodynamic therapy with dobutamine and dopamine to achieve supranormal values for the cardiac index failed to reduce morbidity or mortality among critically ill patients [45]. Michelle et al. also demonstrated that the use of dobutamine to boost the cardiac index did not improve the outcome of critically ill patients [46]. Furthermore, Wilkman et al. reported that the use of dobutamine was associated with increased 90-day mortality from septic shock [47], while Hernandez et al. demonstrated that dobutamine did not improve sublingual microcirculatory, metabolic, hepatosplanchnic, or peripheral perfusion parameters in patients with septic shock [48].

Lyte et al. suggested that the ability of inotropic catecholamines to stimulate bacterial proliferation and biofilm formation might be an etiological factor in the development of intravascular catheter colonization and catheter-related infection [49]. This effect of inotropic catecholamines does not seem to be limited to only coagulase-negative *Staphylococcus* but also other gram-negative bacteria. The growth of these bacteria and production of virulence are associated with inotropic catecholamines [49]. Thus, inotropic catecholamines, such as dobutamine, may have adverse effects in patients with septic shock, and the decrease in β-adrenergic response in patients with sepsis-induced cardiomyopathy may be a protective mechanism to these effects. Morelli et al. suggest that β-blockade could be associated with reductions in the heart rate without adverse effects and that this could help to improve survival [50]. Although the mortality in the control group of their study was high, the study provided interesting preliminary data suggesting that β-blockade may be effective in septic shock treatment. For these reasons, despite the beneficial effects of dobutamine, it appears that excessive increases in sympathetic tone during sepsis can create adverse effects.

Levosimendan can increase contractile myofilament sensitivity to calcium and is a positive inotropic drug. Levosimendan sensitizes troponin C to calcium in a calcium concentration-dependent manner; this increases the effects of calcium on myofilaments during systole. This sensitization is diminished by decreasing calcium concentration level during diastole, and thus, diastolic relaxation remains largely unaffected. In contrast to other inotropic agents, levosimendan does not cause arrhythmias or increase the oxygen consumption. It also opens the ATP-sensitive potassium channels causing smooth muscle membrane hyperpolarization which leads to vasodilation. A meta-analysis evaluated the use of levosimendan in septic shock [51] and reported that it was associated with reduced mortality when compared with standard inotropic therapy. Although levosimendan is also an inotropic agent, it does not stimulate β-adrenergic receptor. This may be the reason why levosimendan can be effective to the patients with septic shock, despite dobutamine seems to create adverse effect in the patients with septic shock. To confirm this finding, a larger multicenter randomized trial is needed to assess the effectiveness of levosimendan in sepsis-induced cardiomyopathy.

Intra-aortic balloon pumping (IABP) is expected to increase the cardiac output and reduce the dosage of a vasopressor. Solomon et al. demonstrated that in a canine model of severe septic shock with a low cardiac index, IABP prolongs survival time and lowers vasopressor requirements [52]. Nakamura et al. reported two cases of severe sepsis-induced cardiomyopathy with refractory shock [53]. To our knowledge, this is the only report in which polymyxin B-immobilized fiber column-direct hemoperfusion (PMX-DHP) and IABP were used for the management of sepsis-induced cardiomyopathy. Although the authors suggested their use in sepsis-induced cardiomyopathy, their effectiveness and safety are not yet developed and their use in the management of septic shock are currently at an experimental stage. For example, a recent multicenter randomized controlled trial demonstrated a non-significant increase in mortality and no improvement in organ failure with PMX-DHP compared to the conventional treatment in patients with septic shock due to peritonitis [54]. There are some case reports of the successful use of veno-arterial extracorporeal membrane oxygenation (ECMO) as the last rescue therapy to unresponsive severe cardiogenic shock in patients with sepsis-induced cardiomyopathy. We searched PubMed (January 1990 to September 2015) for English language articles for sepsis-induced cardiomyopathy treated with veno-arterial ECMO. The keywords “sepsis” OR “septic shock” AND “extracorporeal membrane oxygenation” were used and we carefully reviewed the articles found. The adult patients with sepsis-induced cardiomyopathy who received veno-arterial ECMO support are listed in Table 1 [55–63]. This strategy may provide time for antibiotics to work effectively. If both septic and cardiogenic factors contribute to the pathophysiology of shock, veno-arterial ECMO may improve the mortality of the most severe group. However, the experience of the use of ECMO in patients with septic shock is very limited. Moreover, the management of patients who need ECMO is very complex; thus, a well-experienced team should use it in a specialized center [64]. For these reasons, to date, mechanical support with IABP or ECMO seems not to be a standard therapy, although it may be the last option for unresponsive severe cardiogenic shock due to sepsis-induced cardiomyopathy.

**Table 1:** Reported cases of the patients with sepsis-induced cardiomyopathy who received veno-arterial extracorporeal membrane oxygenation support

Article	Age	Sex	Infection	Survival
Pořízka M et al. 2015 [55]	31	M	Necrotizing fasciitis	+
Fujisaki N et al.2014 [56]	27	F	CA pneumonia	+
Endo A et al. 2014 [57]	41	M	Purpura fulminans	−
Bréchot N et al. 2013 [58]	33	M	CA pneumonia	+
Bréchot N et al. 2013 [58]	62	M	CA pneumonia	+
Bréchot N et al. 2013 [58]	31	F	Acute cholecystitis	+
Bréchot N et al. 2013 [58]	33	F	Aspiration pneumonia	+
Bréchot N et al. 2013 [58]	48	F	CA pneumonia	−
Bréchot N et al. 2013 [58]	66	M	Peritonitis after liver transplant	−
Bréchot N et al. 2013 [58]	59	M	CA pneumonia	+
Bréchot N et al. 2013 [58]	52	M	CA pneumonia	−
Bréchot N et al. 2013 [58]	28	F	CA pneumonia	+
Bréchot N et al. 2013 [58]	35	M	Aspiration pneumonia	+
Bréchot N et al. 2013 [58]	28	F	Aspiration pneumonia	+
Bréchot N et al. 2013 [58]	52	F	Nosocomial pneumonia	+
Bréchot N et al. 2013 [58]	57	F	Pharyngitis	+
Bréchot N et al. 2013 [58]	48	M	CA pneumonia	−
Hagiwara et al. 2013 [59]	69	M	Klebsiella bacteremia	+
Firstenberg MS et al. 2010 [60]	18	M	Necrotizing fasciitis	+
Firstenberg MS et al. 2010 [60]	39	F	Necrotizing fasciitis	+
MacLaren G et al. 2010 [61]	29	F	H1N1 influenza	+
Vohra HA et al. 2009 [62]	18	M	Mediastinitis after Ravitch procedure	+
McLauren G et al. 2004 [63]	22	M	Vertebral osteomyelitis	+

*CA* community acquired, *M* male, *F* female

## Summary of our current knowledge

Sepsis-induced cardiomyopathy is characterized by left ventricular dilatation and depressed ejection fraction that typically normalize within 7–10 days. Our current understanding is that sepsis-induced cardiomyopathy is induced by endotoxins and cytokines and that the initial management should be the same as for septic shock without cardiomyopathy. However, the lack of quality evidence that dobutamine improves survival and the concerning reports that it may adversely affect outcomes in patients with sepsis imply that the routine use of dobutamine should no longer be recommended. In the near future, levosimendan or mechanical support with ECMO may be developed as a therapeutic option, but further study is needed to confirm whether it is truly effective in sepsis-induced cardiomyopathy.

## Abbreviations

ECMO: extracorporeal membrane oxygenation
IABP: intra-aortic balloon pumping
PMX-DHP: polymyxin B-immobilized fiber column-direct hemoperfusion
TNF: tumor necrosis factor
